# Crystal structure of 1-methylimidazole 3-oxide monohydrate

**DOI:** 10.1107/S2056989017002079

**Published:** 2017-02-14

**Authors:** Christopher S. Frampton, James I. Murray, Alan C. Spivey

**Affiliations:** a Wolfson Centre for Materials Processing, Brunel University London, Kingston Lane, Uxbridge, UB8 3PH, UK; b Department of Chemistry, South Kensington Campus, Imperial College London, London, SW7 2AZ, UK

**Keywords:** crystal structure, catalysis, aryl *N*-oxides, 1-methyl-2*H*-imidazole 3-*N*-oxide, hydrate, hydrogen bonding

## Abstract

1-Methylimidazole 3-*N*-oxide (NMI-O) crystallizes as a monohydrate, C_4_H_6_N_2_O·H_2_O, in the monoclinic space group *P*2_1_ with *Z*′ = 2 (mol­ecules *A* and *B*). The imidazole rings display a planar geometry (r.m.s. deviations = 0.0008 and 0.0002 Å) and are linked in the crystal structure into infinite zigzag strands of ⋯NMI-O(*A*)⋯OH_2_⋯NMI-O(*B*)⋯OH_2_⋯ units by O—H⋯O hydrogen bonds. These chains propagate along the *b*-axis direction of the unit cell.

## Abstract 2

1-Methylimidazole 3-*N*-oxide (NMI-O) crystallizes as a monohydrate, in the monoclinic space group *P*2_1_ with *Z*′ = 2 (mol­ecules *A* and *B*). The imidazole rings display a planar geometry and are linked in the crystal structure into infinite zigzag strands of ⋯NMI-O(*A*)⋯OH_2_⋯NMI-O(*B*)⋯OH_2_⋯ units by O—H⋯O hydrogen bonds. These chains propagate along the *b*-axis direction of the unit cell.

## Chemical context   

Aryl-*N*-oxides are an important class of materials acting as highly efficient catalysts for the phospho­rylation of alcohols (Murray *et al.*, 2015 ▸) and also for the site-selective phos­phoyl­ation of polyols and peptides (Murray *et al.* 2014 ▸). One material in particular, 1-methylimidazole 3-*N*-oxide, (NMI-O), has been shown to be a highly efficient catalyst for both sulfonyl­ation and silylation procedures (Murray & Spivey, 2015 ▸). Until recently, NMI-O has been somewhat elusive in the literature. The synthesis of NMI-O and its use as a highly efficient catalyst for certain Morita–Baylis–Hillman reactions has been reported (Lin *et al.*, 2005 ▸) although no conclusive information on the structural identity of the material synthesized was presented. A recent paper, directed at the synthesis of salts of 1-alkyl-imidazole 3-oxides for use as ionic liquids also reported the synthesis of NMI-O, however all attempts at crystallizing a sample of this material were unsuccessful although two crystalline adducts of NMI-O, a tris (2-thien­yl)borane and a silver carbene hexa­fluorido­phosphate, were structurally characterized (Laus *et al.*, 2008 ▸). These authors also demonstrated by NMR and subsequent X-ray structural analysis of a related 1,2-di­methyl­imidazole semiperhydrate material that the likely product reported earlier (Lin *et al.*, 2005 ▸) was the 1-methylimidazole semiperhydrate rather than NMI-O itself. We now present a simplified synthesis of MNI-O and the crystal structure of its hydrate.
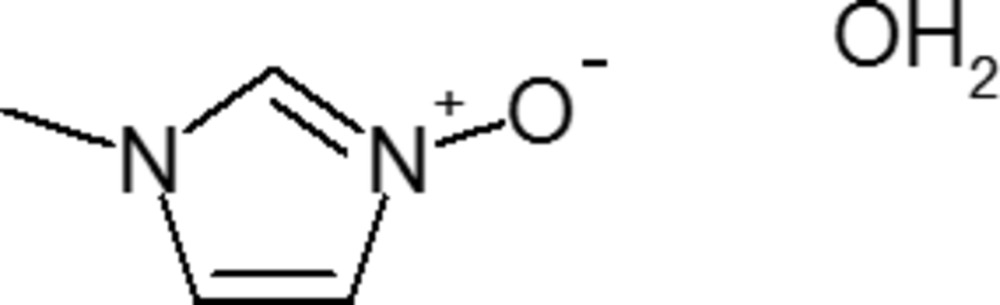


## Structural commentary   

**Figure 1:**
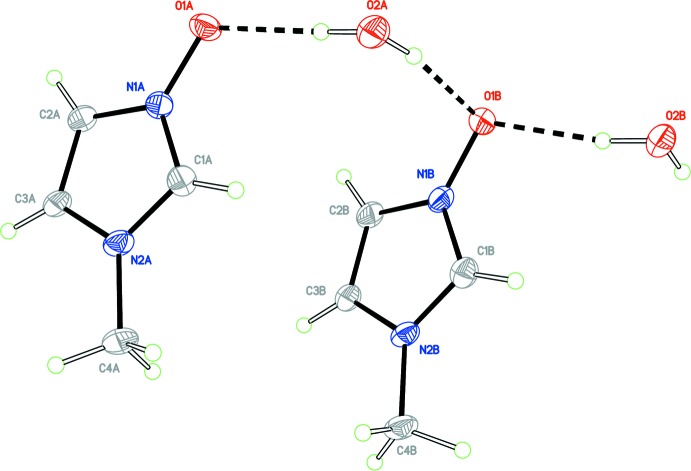
View of the asymmetric unit of the title compound with the atom labelling. Displacement ellipsoids are drawn at the 50% probability level. The O—H⋯O hydrogen bonds are shown as dashed lines.

The asymmetric unit of the title compound is shown in Fig. 1 ▸. It contains two mol­ecules of NMI-O and two fully occupied and ordered water mol­ecules, making the overall stoichiometry a monohydrate. A calculated least-squares plane through the five atoms of the imidazole ring (C1, N1, C2, C3, N2) for mol­ecules *A* and *B* gave r.m.s. deviations from planarity of 0.0008 and 0.0002 Å, respectively, with the oxygen atoms of the N^+^— O^−^ groups also residing close to the ring plane; O1*A*, −0.021 (4) Å; O1*B*, −0.008 (4) Å. The methyl groups lie somewhat farther outside the plane of the ring with displacements of −0.073 (5) Å for C4*A* and −0.116 (1) Å for C4*B*. The dihedral angle formed between the least-squares planes of the *A* and *B* NMI-O mol­ecules is 12.96 (16)°. The present data were not of sufficient quality to determine the absolute structure.

## Supra­molecular features   

**Figure 2:**
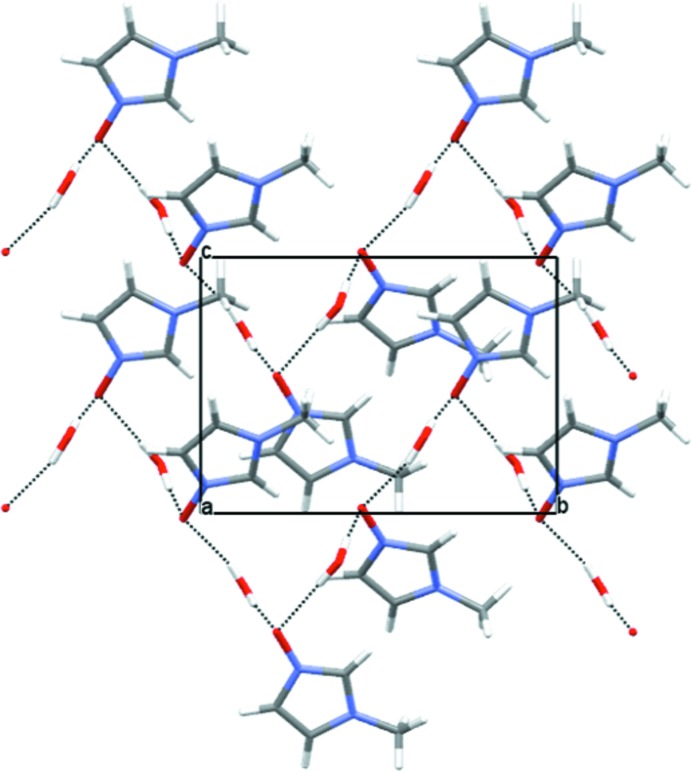
View of the crystal packing down the *a* axis. The O—H⋯O hydrogen bonds (see Table 1 ▸) are shown as dotted lines.

In the crystal, the NMI-O and water mol­ecules are linked by O—H⋯O hydrogen bonds to form an infinite NMI-O⋯OH_2_⋯NMI-O⋯OH_2_⋯ chain propagating along the *b*-axis direction of the unit cell. Each water mol­ecule forms two hydrogen bonds, one to each of the N^+^— O^−^ groups of NMI-O mol­ecules *A* and *B* with the oxygen atoms of these groups acting as double acceptors from both water mol­ecules (Table 1 ▸, Fig. 2 ▸). The NMI-O⋯OH_2_⋯NMI-O⋯OH_2_⋯ chains are cross-linked in the crystal structure by weaker C—H⋯O inter­actions (Table 1 ▸) with H⋯O contacts in the range 2.41–2.56 Å.

## Database survey   

A search of the Cambridge Structural Database (CSD, Version 5.37 update February 2016; Groom *et al.*, 2016 ▸) for the imidazole-3-oxide substructure yielded 16 hits, all of which were genuine examples of substituted imidazole-3-oxides. Closely related examples include 1-hy­droxy­imidazole-3-oxide (DOJKUJ), 1-hy­droxy-2-methyl­imidazole-3-oxide (DOJLAQ), 3-hy­droxy-1,2-di­methyl­imidazolium 1,2-di­meth­yl­imidazolium-3-oxide iodide (DOJMUL) and 1,2-di­methyl­imidazole-3-oxide (DOJNAS) (Laus *et al.*, 2008 ▸). For 1-hy­droxy-2,4,5-triphenyl-1*H*-imidazole 3-oxide (JADNAE; Sánchez-Migallón *et al.* 2003 ▸), the N^+^— O^−^ bond length was particularly short at 1.276 and 1.278 Å for the two mol­ecules in the asymmetric unit. For the title compound, the N^+^—O^−^ bond lengths are 1.350 (3) and 1.348 (3)Å for mol­ecules *A* and *B*, respectively. These values are within the range exhibited for the remaining 15 database entries (1.326–1.368 Å).

## Synthesis and crystallization   

The title compound was synthesized in a three-step, one-pot process in which aqueous glyoxal was condensed with hydroxyl­amine hydro­chloride in the presence of sodium carbonate to afford the mono-oxime. This inter­mediate was immediately condensed with methyl­amine to give the corres­ponding imine, which cyclo-condenses upon exposure to aqueous formaldehyde to give NMI-O after acidic workup in ∼68% yield (Murray & Spivey, 2016 ▸). The previously reported synthesis also started from glyoxal but required eight steps (Laus *et al.*, 2008 ▸). The material was concentrated *in vacuo* to afford a brown oil, which crystallized overnight as colourless laths in the freezer after exposure to air, forming a monohydrate species. The crystals as prepared were extremely hygroscopic, necessitating a rapid transfer to the cold stream of the diffractometer.

## Refinement   

Crystal data, data collection and structure refinement details are summarized in Table 2 ▸. The four water H atoms were located in a Fourier difference map and freely refined. All the remaining H atoms were placed geometrically in idealized positions and allowed to ride on their parent atoms: C—H = 0.95–0.98Å with *U*_iso_(H) = 1.5*U*_eq_(C-meth­yl) and *U*_iso_(H) = 1.2*U*_eq_(C) for other H atoms. The data were not of a sufficient quality to reliably determine the absolute structure.

## Supplementary Material

Crystal structure: contains datablock(s) I. DOI: 10.1107/S2056989017002079/hb7643sup1.cif

Structure factors: contains datablock(s) I. DOI: 10.1107/S2056989017002079/hb7643Isup2.hkl

Supporting information file. DOI: 10.1107/S2056989017002079/hb7643Isup3.cml

CCDC reference: 1531714

Additional supporting information:  crystallographic information; 3D view; checkCIF report

## Figures and Tables

**Table 1:** Hydrogen-bond geometry (Å, °)

*D*—H⋯*A*	*D*—H	H⋯*A*	*D*⋯*A*	*D*—H⋯*A*
O2*A*—H2*AA*⋯O1*B*	1.03 (6)	1.73 (6)	2.752 (3)	172 (4)
O2*A*—H2*AB*⋯O1*A*	0.83 (5)	1.94 (5)	2.773 (3)	175 (4)
O2*B*—H2*BA*⋯O1*B*	0.83 (4)	1.94 (4)	2.752 (3)	167 (4)
O2*B*—H2*BB*⋯O1*A* ^i^	0.94 (5)	1.86 (5)	2.790 (3)	171 (5)
C1*A*—H1*A*⋯O1*A* ^ii^	0.95	2.47	3.248 (4)	139
C4*A*—H4*AC*⋯O1*A* ^ii^	0.98	2.46	3.308 (4)	145
C4*B*—H4*BC*⋯O1*A* ^ii^	0.98	2.56	3.336 (4)	136
C1*B*—H1*B*⋯O1*B* ^i^	0.95	2.48	3.248 (4)	138
C2*B*—H2*B*⋯O2*B* ^iii^	0.95	2.41	3.298 (4)	155
C4*B*—H4*BA*⋯O1*B* ^i^	0.98	2.50	3.345 (4)	144

Symmetry codes: (i) 
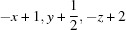
; (ii) 
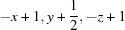
; (iii) 
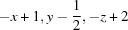
.

**Table 2:** Experimental details

Crystal data	
Chemical formula	C_4_H_6_N_2_O·H_2_O
*M*_r_	116.12
Crystal system, space group	Monoclinic, *P*2_1_
Temperature (K)	100
*a*, *b*, *c* (Å)	7.5941 (6), 10.0703 (6), 7.8286 (6)
β (°)	112.402 (9)
*V* (Å^3^)	553.51 (8)
*Z*	4
Radiation type	Cu *K*α
μ (mm^−1^)	0.95
Crystal size (mm)	0.45 × 0.10 × 0.05

Data collection	
Diffractometer	Rigaku SuperNova, Dualflex, AtlasS2
Absorption correction	Multi-scan (*CrysAlis PRO*; Rigaku OD, 2015 ▸)
*T*_min_, *T*_max_	0.419, 1.000
No. of measured, independent and observed [*I* > 2σ(*I*)] reflections	2067, 1386, 1241
*R*_int_	0.023
(sin θ/λ)_max_ (Å^−1^)	0.624

Refinement	
*R*[*F* ^2^ > 2σ(*F* ^2^)], *wR*(*F* ^2^), *S*	0.042, 0.119, 1.01
No. of reflections	1386
No. of parameters	163
No. of restraints	1
H-atom treatment	H atoms treated by a mixture of independent and constrained refinement
Δρ_max_, Δρ_min_ (e Å^−3^)	0.21, −0.23

Computer programs: *CrysAlis PRO* (Rigaku OD, 2015 ▸), *SHELXD2014* (Sheldrick *et al.*, 2001 ▸), *SHELXL2014* (Sheldrick, 2015 ▸), *SHELXTL* (Sheldrick, 2008 ▸), *Mercury* (Macrae *et al.*, 2008 ▸) and *publCIF* (Westrip, 2010 ▸).

## supplementary crystallographic information

### Crystal data

**Table:**

C_4_H_6_N_2_O·H_2_O	*F*(000) = 248
*M**_r_* = 116.12	*D*_x_ = 1.393 Mg m^−^^3^
Monoclinic, *P*2_1_	Cu *K*α radiation, λ = 1.54184 Å
*a* = 7.5941 (6) Å	Cell parameters from 1007 reflections
*b* = 10.0703 (6) Å	θ = 6.3–74.8°
*c* = 7.8286 (6) Å	µ = 0.95 mm^−^^1^
β = 112.402 (9)°	*T* = 100 K
*V* = 553.51 (8) Å^3^	Lath, colourless
*Z* = 4	0.45 × 0.10 × 0.05 mm

### Data collection

**Table:**

Rigaku SuperNova, Dualflex, AtlasS2 diffractometer	1386 independent reflections
Radiation source: fine-focus sealed X-ray tube, Enhance (Cu) X-ray Source	1241 reflections with *I* > 2σ(*I*)
Detector resolution: 5.2921 pixels mm^-1^	*R*_int_ = 0.023
ω scans	θ_max_ = 74.3°, θ_min_ = 6.1°
Absorption correction: multi-scan (CrysAlis PRO; Rigaku OD, 2015)	*h* = −9→8
*T*_min_ = 0.419, *T*_max_ = 1.000	*k* = −12→5
2067 measured reflections	*l* = −9→7

### Refinement

**Table:**

Refinement on *F*^2^	Primary atom site location: structure-invariant direct methods
Least-squares matrix: full	Hydrogen site location: mixed
*R*[*F*^2^ > 2σ(*F*^2^)] = 0.042	H atoms treated by a mixture of independent and constrained refinement
*wR*(*F*^2^) = 0.119	*w* = 1/[σ^2^(*F*_o_^2^) + (0.075*P*)^2^] where *P* = (*F*_o_^2^ + 2*F*_c_^2^)/3
*S* = 1.01	(Δ/σ)_max_ < 0.001
1386 reflections	Δρ_max_ = 0.21 e Å^−^^3^
163 parameters	Δρ_min_ = −0.23 e Å^−^^3^
1 restraint	

### Special details

**Table:**

Geometry. All esds (except the esd in the dihedral angle between two l.s. planes) are estimated using the full covariance matrix. The cell esds are taken into account individually in the estimation of esds in distances, angles and torsion angles; correlations between esds in cell parameters are only used when they are defined by crystal symmetry. An approximate (isotropic) treatment of cell esds is used for estimating esds involving l.s. planes.

### Fractional atomic coordinates and isotropic or equivalent isotropic displacement parameters (Å^2^)

**Table:**

	*x*	*y*	*z*	*U*_iso_*/*U*_eq_	
O1A	0.5193 (3)	0.2133 (2)	0.5360 (3)	0.0218 (5)	
N1A	0.3842 (3)	0.2757 (3)	0.3934 (3)	0.0176 (6)	
N2A	0.2057 (4)	0.4269 (3)	0.2161 (4)	0.0189 (6)	
C1A	0.3553 (4)	0.4046 (3)	0.3747 (4)	0.0193 (6)	
H1A	0.4276	0.4707	0.4589	0.023*	
C2A	0.2517 (4)	0.2107 (3)	0.2439 (4)	0.0185 (6)	
H2A	0.2413	0.1176	0.2231	0.022*	
C3A	0.1392 (4)	0.3066 (3)	0.1325 (4)	0.0185 (6)	
H3A	0.0348	0.2931	0.0187	0.022*	
C4A	0.1200 (4)	0.5563 (3)	0.1484 (5)	0.0231 (7)	
H4AA	0.0184	0.5739	0.1936	0.035*	
H4AB	0.0667	0.5562	0.0130	0.035*	
H4AC	0.2176	0.6256	0.1934	0.035*	
O2A	0.7412 (3)	0.3868 (2)	0.8081 (3)	0.0237 (5)	
H2AA	0.675 (7)	0.410 (6)	0.898 (7)	0.063 (16)*	
H2AB	0.669 (6)	0.335 (5)	0.729 (6)	0.039 (13)*	
O1B	0.5360 (3)	0.4513 (2)	1.0200 (3)	0.0226 (5)	
N1B	0.3895 (4)	0.5126 (3)	0.8873 (3)	0.0188 (6)	
N2B	0.2080 (4)	0.6632 (3)	0.7115 (4)	0.0183 (6)	
C1B	0.3733 (4)	0.6430 (3)	0.8560 (4)	0.0203 (7)	
H1B	0.4617	0.7092	0.9231	0.024*	
C2B	0.2336 (4)	0.4478 (3)	0.7620 (4)	0.0201 (6)	
H2B	0.2105	0.3548	0.7544	0.024*	
C3B	0.1191 (4)	0.5435 (3)	0.6509 (4)	0.0194 (6)	
H3B	0.0007	0.5300	0.5510	0.023*	
C4B	0.1431 (4)	0.7915 (3)	0.6217 (5)	0.0222 (7)	
H4BA	0.1842	0.8624	0.7144	0.033*	
H4BB	0.0038	0.7916	0.5622	0.033*	
H4BC	0.1978	0.8065	0.5284	0.033*	
O2B	0.7272 (3)	0.6268 (2)	1.2987 (3)	0.0242 (6)	
H2BA	0.663 (6)	0.585 (5)	1.205 (5)	0.023 (10)*	
H2BB	0.638 (7)	0.648 (6)	1.351 (7)	0.057 (15)*	

### Atomic displacement parameters (Å^2^)

**Table:**

	*U*^11^	*U*^22^	*U*^33^	*U*^12^	*U*^13^	*U*^23^
O1A	0.0226 (10)	0.0189 (12)	0.0202 (12)	0.0033 (9)	0.0039 (10)	0.0048 (10)
N1A	0.0212 (12)	0.0148 (13)	0.0171 (12)	0.0005 (9)	0.0075 (10)	0.0015 (10)
N2A	0.0236 (12)	0.0108 (14)	0.0229 (13)	0.0007 (10)	0.0095 (11)	0.0008 (10)
C1A	0.0210 (13)	0.0169 (16)	0.0196 (15)	−0.0029 (12)	0.0071 (12)	−0.0001 (11)
C2A	0.0228 (14)	0.0103 (15)	0.0223 (15)	−0.0013 (12)	0.0084 (13)	−0.0007 (12)
C3A	0.0227 (14)	0.0101 (14)	0.0219 (15)	−0.0012 (11)	0.0077 (12)	−0.0025 (12)
C4A	0.0290 (15)	0.0107 (15)	0.0300 (16)	0.0013 (12)	0.0117 (14)	0.0027 (13)
O2A	0.0248 (11)	0.0177 (13)	0.0263 (12)	−0.0010 (9)	0.0071 (10)	−0.0023 (10)
O1B	0.0256 (11)	0.0192 (11)	0.0185 (10)	0.0039 (9)	0.0032 (9)	−0.0010 (9)
N1B	0.0243 (13)	0.0137 (14)	0.0187 (13)	0.0015 (10)	0.0086 (11)	−0.0017 (9)
N2B	0.0229 (12)	0.0110 (13)	0.0220 (12)	0.0005 (10)	0.0097 (10)	−0.0001 (10)
C1B	0.0230 (15)	0.0181 (16)	0.0209 (14)	−0.0025 (12)	0.0097 (13)	−0.0020 (12)
C2B	0.0263 (15)	0.0111 (14)	0.0224 (14)	−0.0015 (12)	0.0087 (12)	−0.0012 (11)
C3B	0.0217 (13)	0.0139 (15)	0.0210 (13)	−0.0024 (12)	0.0063 (12)	−0.0032 (12)
C4B	0.0280 (15)	0.0108 (15)	0.0279 (16)	0.0015 (13)	0.0107 (14)	0.0025 (12)
O2B	0.0265 (12)	0.0200 (14)	0.0251 (11)	−0.0013 (10)	0.0086 (10)	−0.0053 (10)

### Geometric parameters (Å, º)

**Table:**

O1A—N1A	1.350 (3)	O1B—N1B	1.348 (3)
N1A—C1A	1.315 (4)	N1B—C1B	1.332 (4)
N1A—C2A	1.384 (4)	N1B—C2B	1.380 (4)
N2A—C1A	1.344 (4)	N2B—C1B	1.348 (4)
N2A—C3A	1.378 (4)	N2B—C3B	1.374 (4)
N2A—C4A	1.463 (4)	N2B—C4B	1.464 (4)
C1A—H1A	0.9500	C1B—H1B	0.9500
C2A—C3A	1.362 (4)	C2B—C3B	1.366 (4)
C2A—H2A	0.9500	C2B—H2B	0.9500
C3A—H3A	0.9500	C3B—H3B	0.9500
C4A—H4AA	0.9800	C4B—H4BA	0.9800
C4A—H4AB	0.9800	C4B—H4BB	0.9800
C4A—H4AC	0.9800	C4B—H4BC	0.9800
O2A—H2AA	1.03 (6)	O2B—H2BA	0.83 (4)
O2A—H2AB	0.83 (5)	O2B—H2BB	0.94 (5)
			
C1A—N1A—O1A	126.5 (3)	C1B—N1B—O1B	125.7 (3)
C1A—N1A—C2A	109.6 (3)	C1B—N1B—C2B	110.0 (3)
O1A—N1A—C2A	123.9 (3)	O1B—N1B—C2B	124.3 (3)
C1A—N2A—C3A	108.6 (3)	C1B—N2B—C3B	109.6 (3)
C1A—N2A—C4A	125.8 (3)	C1B—N2B—C4B	124.9 (3)
C3A—N2A—C4A	125.4 (3)	C3B—N2B—C4B	125.3 (3)
N1A—C1A—N2A	108.3 (3)	N1B—C1B—N2B	107.1 (3)
N1A—C1A—H1A	125.8	N1B—C1B—H1B	126.4
N2A—C1A—H1A	125.8	N2B—C1B—H1B	126.4
C3A—C2A—N1A	106.4 (3)	C3B—C2B—N1B	106.5 (3)
C3A—C2A—H2A	126.8	C3B—C2B—H2B	126.7
N1A—C2A—H2A	126.8	N1B—C2B—H2B	126.7
C2A—C3A—N2A	107.0 (3)	C2B—C3B—N2B	106.8 (3)
C2A—C3A—H3A	126.5	C2B—C3B—H3B	126.6
N2A—C3A—H3A	126.5	N2B—C3B—H3B	126.6
N2A—C4A—H4AA	109.5	N2B—C4B—H4BA	109.5
N2A—C4A—H4AB	109.5	N2B—C4B—H4BB	109.5
H4AA—C4A—H4AB	109.5	H4BA—C4B—H4BB	109.5
N2A—C4A—H4AC	109.5	N2B—C4B—H4BC	109.5
H4AA—C4A—H4AC	109.5	H4BA—C4B—H4BC	109.5
H4AB—C4A—H4AC	109.5	H4BB—C4B—H4BC	109.5
H2AA—O2A—H2AB	107 (4)	H2BA—O2B—H2BB	103 (4)
			
O1A—N1A—C1A—N2A	−178.9 (2)	O1B—N1B—C1B—N2B	179.6 (2)
C2A—N1A—C1A—N2A	0.2 (3)	C2B—N1B—C1B—N2B	0.0 (4)
C3A—N2A—C1A—N1A	−0.1 (3)	C3B—N2B—C1B—N1B	0.0 (3)
C4A—N2A—C1A—N1A	176.4 (3)	C4B—N2B—C1B—N1B	−174.4 (3)
C1A—N1A—C2A—C3A	−0.2 (3)	C1B—N1B—C2B—C3B	0.0 (4)
O1A—N1A—C2A—C3A	179.0 (2)	O1B—N1B—C2B—C3B	−179.6 (2)
N1A—C2A—C3A—N2A	0.1 (3)	N1B—C2B—C3B—N2B	0.0 (3)
C1A—N2A—C3A—C2A	0.0 (3)	C1B—N2B—C3B—C2B	0.0 (3)
C4A—N2A—C3A—C2A	−176.6 (3)	C4B—N2B—C3B—C2B	174.4 (3)

### Hydrogen-bond geometry (Å, º)

**Table:**

*D*—H···*A*	*D*—H	H···*A*	*D*···*A*	*D*—H···*A*
O2*A*—H2*AA*···O1*B*	1.03 (6)	1.73 (6)	2.752 (3)	172 (4)
O2*A*—H2*AB*···O1*A*	0.83 (5)	1.94 (5)	2.773 (3)	175 (4)
O2*B*—H2*BA*···O1*B*	0.83 (4)	1.94 (4)	2.752 (3)	167 (4)
O2*B*—H2*BB*···O1*A*^i^	0.94 (5)	1.86 (5)	2.790 (3)	171 (5)
C1*A*—H1*A*···O1*A*^ii^	0.95	2.47	3.248 (4)	139
C4*A*—H4*AC*···O1*A*^ii^	0.98	2.46	3.308 (4)	145
C4*B*—H4*BC*···O1*A*^ii^	0.98	2.56	3.336 (4)	136
C1*B*—H1*B*···O1*B*^i^	0.95	2.48	3.248 (4)	138
C2*B*—H2*B*···O2*B*^iii^	0.95	2.41	3.298 (4)	155
C4*B*—H4*BA*···O1*B*^i^	0.98	2.50	3.345 (4)	144

Symmetry codes: (i) −*x*+1, *y*+1/2, −*z*+2; (ii) −*x*+1, *y*+1/2, −*z*+1; (iii) −*x*+1, *y*−1/2, −*z*+2.
